# Potential for Bacteriophage Cocktail to Complement Commercial Sanitizer Use on Produce against *Escherichia coli* O157:H7

**DOI:** 10.3390/microorganisms8091316

**Published:** 2020-08-29

**Authors:** Badrinath Vengarai Jagannathan, Steven Kitchens, Paul Priyesh Vijayakumar, Stuart Price, Melissa Morgan

**Affiliations:** 1 Department of Animal and Food Sciences, University of Kentucky, Lexington, KY 40456, USA; badrivj@uky.edu (B.V.J.); paul.v@uky.edu (P.P.V.); 2 Department of Pathobiology, College of Veterinary Medicine, Auburn University, AL 36849, USA; srk0002@auburn.edu (S.K.); pricesb@auburn.edu (S.P.)

**Keywords:** bacteriophage, characterization, *E. coli*, sanitizer, microplate

## Abstract

The increasing concern for food safety has created a need to evaluate novel techniques to eliminate or control pathogens, resulting in safe food. In this study, four bacteriophages of bovine origin, specific to *E. coli* O157:H7, were successfully isolated and characterized. A microplate reader assay demonstrated the efficacy of the bacteriophage (phage) cocktail against *E. coli* O157:H7 resulting in a significant reduction (*p* < 0.01) in the target pathogen population. The phage cocktail demonstrated significant efficacy (*p* < 0.05) against *E. coli* O157:H7 in the presence of the most utilized sanitizers in the United States, namely 100 parts per million (ppm) free chlorine and 100-ppm peroxyacetic acid. Survival in the sanitizer concentrations demonstrates the potential use of phage cocktail and sanitizer synergistically to enhance sanitation operations in the food industry.

## 1. Introduction

Foodborne illnesses of microbial origin can range from mild to life-threatening, depending on the source and type of contamination. Numerous outbreaks linked to contaminated fruits and vegetables have emerged in recent years [1]. Outbreaks, particularly associated with raw produce, are a significant concern because produce is consumed raw and is more likely to harbor foodborne pathogens [2]. Several environmental factors contribute to contaminating fresh produce with spoilage and pathogenic microorganisms during pre- and post-harvest processing [3]. These pathogenic microorganisms include *Campylobacter* spp., enterotoxigenic *Bacillus cereus*, *Escherichia coli* O157:H7 and other Shiga toxin-producing *E. coli*, *Listeria monocytogenes*, *Salmonella* spp., *Shigella* spp., enterotoxigenic *Staphylococcus aureus*, certain viruses, and protozoa [2]. Among those listed above, a certain strain of *Escherichia coli* (*E. coli*), serotype O157:H7, is a significant pathogen that contaminates fresh produce and is among the leading cause of foodborne outbreaks of gastroenteritis. Although Shiga toxin-producing *E. coli* illness is often associated with beef consumption, several outbreaks have been traced back to the consumption of contaminated sprouts and pre-packaged spinach [4].

Traditionally, pathogen control on produce in the United States has relied on using chemical sanitizers such as chlorine and peroxyacetic acid. Wash water with a concentration maximum of 200 parts per million (ppm) of free chlorine and 100 ppm of peroxyacetic acid is commonly recommended and used in the United States for washing fresh produce [5,6,7]. Although these recommendations are followed in the industry, outbreaks related to fresh produce still occur. Yu et al. (2001) studied the effectiveness of chemical agents on reducing *E. coli* O157:H7-contaminated strawberry fruit. The researchers employed different chemical agents such as bleach (100–200 ppm), hydrogen peroxide (1 and 3%), Tween 80 (100 and 200 ppm), and acetic acid (2–5%) to study their effectiveness in reducing the population of the pathogen. None of the chemical agents, except hydrogen peroxide, achieved more than a 2-log reduction of the pathogen on the surface of the fruits [8].

Antibiotics historically treat bacterial infections; however, severe medical and social problems have emerged due to the development of antibiotic-resistant bacteria strains [9]. Before the discovery and prevalent use of antibiotics, bacteriophages were considered for the prevention or treatment of various bacterial infections [10]. Bacteriophages, informally known as phage, are bacterial viruses that invade and replicate within bacteria and, in the case of the lytic phage, disrupt bacterial metabolism that causes the bacterium to lyse [10]. Historically, the study of phages suffered from conflicting observations, misinterpretation, and incomplete understanding. Currently, phages are being used in the food industry, due to their antimicrobial potential [11,12]. In the last decade, there has been an increasing number of regulatory approvals issued for phage preparations, especially for the ones used for improving food safety [13]. In 2006, the first phage preparation (ListShield™) was utilized in the United States against *Listeria monocytogenes* as a direct application on fish and shellfish, fresh and processed fruits, fresh and processed vegetables, and dairy products (including cheese) [13,14]. In recent years, several phage products have been granted GRAS (Generally Recognized as Safe) approval by FDA (Food and Drug Administration) and are now commercially available in the market to improve the safety of food products. In line with the clearance of phage treatments by the United States, several other countries such as Canada, Switzerland, Australia, and the European Union, have also issued approvals for phage preparations to be successfully used in the food industry [13].

The fresh produce retail industries have increased their production of pre-packaged salad and fruit [4]. As a result, there is a parallel increase in foodborne outbreaks linked to fresh produce [4]. Due to the increase in foodborne outbreaks caused by these pathogens, it appears that current technologies employed to stop bacterial pathogens in the food industry are not reliable [15]. Due to the delicate nature and raw consumption of fresh fruits and vegetables, some approaches traditionally used in the food industry to reduce contamination by pathogens are not appropriate. Hence, despite recent advances to avoid transmission of bacterial pathogens throughout the food chain, novel strategies are still required to fulfill consumer demands for minimally processed foods with fewer chemical preservatives [15]. Optical density measurement, using a microplate reader, is a technique used to determine the inhibitory effects of antimicrobial agents obtained from plants, spices, and other foods [16]. Knezevic and Petrovic (2008) used a microplate technique with crystal violet staining and optical density measurements to evaluate *Pseudomonas aeruginosa* bacteriophage’s ability to inhibit and eradicate biofilm formation [17].

The first objective of the current study was to isolate bacteriophages of bovine origin specific to *E. coli* O157:H7 and evaluate their ability, in a cocktail, to infect and kill pathogenic *E. coli* O15:H7, thus, controlling the contamination of the pathogen. The second objective was to determine the potential synergistic effect of using bacteriophages combined with commercial sanitizers such as chlorine and peroxyacetic acid (SaniDate 5.0) at 100-ppm (parts per million) concentration to reduce *E. coli* O157:H7 contamination.

## 2. Materials and Methods

### 2.1. Bacteriophage Screening, Purification, and Amplification

Bacteriophages were isolated from the environment by taking a swab of bovine feces collected from the Auburn University College of Veterinary Medicine dairy herd pastures and placing it in brain heart infusion broth (BHI; Bacto Brain Heart Infusion, Becton Dickinson, Sparks, MD, USA) containing 20 µg/mL novobiocin and 2.5 µg/mL potassium tellurite. After incubation overnight at 37 degrees Celsius (°C), 1 mL of the bacterial suspension in the broth was centrifuged at 12,500 times gravity (×*g*) for 15 min, and the resulting supernatant was filter sterilized through a 0.2 µm filter (Sterile Syringe Filter with 0.2 µm Polyethersulfone Membrane, VWR International). To generate phage plaques, a bacterial lawn of *Escherichia coli* (*E. coli*) O157:H7 (ATCC 43895) prepared by culturing the strain in a bacteriological incubator with aeration at 37 °C to log phase in Luria-Bertani broth (LB; Difco LB Broth, Miller, Becton Dickinson, Sparks, MD, USA) containing 1 mM magnesium (LBM). The media was then diluted to an absorbance, optical density, and measured at a wavelength of 620 nm (OD_620_) of 0.8 to 1.0. *E. coli* (ATCC 43895) (0.2 mL). The diluted media containing the *E. coli* (ATCC 43895) was then mixed with the phage supernatant, incubated at 37 °C for 20 min to allow phage adsorption to the cells, and mixed with 3.0 mL of molten soft agar (LBM with 0.7% Bacto agar). The molten LBM soft agar with *E. coli* (ATCC 43895) and the supernatant was poured onto the LBM underlay, or bottom agar plates (LBM with 1.5% agar-agar), using the double agar overlay technique [18]. The plates were allowed to solidify for one hour then incubated overnight at 37 °C. Two plaques were cored using a sterile Pasteur pipette from each plate that showed plaque formation, and placed in 0.5 mL salts-magnesium (SM) buffer at 5 °C, for at least 5 h for [18].

For bacteriophage plaque purification, *E. coli* (ATCC 43895) cells were cultured to log phase, then diluted to an OD_620_ of 0.8 to 1.0. Serial dilutions of each bacteriophage solution were performed, and 0.2 mL of the *E. coli* (ATCC 43895) cells were mixed with 10 µL of the bacteriophage solution. The cells were incubated with the bacteriophage for twenty minutes before adding 3 mL soft agar and pouring the mixture onto an LBM agar plate. The plates were allowed to solidify and were incubated overnight at 37 °C. Isolated bacteriophage plaques were cored, and the cores were placed in 0.5 mL SM buffer, stored at 5 °C, and allowed to diffuse for at least 5 h. The plaque purification procedure was repeated in order to achieve a pure culture of the bacteriophage.

For the production of high titer stocks, 50 mL of log-phase *E. coli* (ATCC 43895) cells grown in LBM broth was inoculated with 0.5 mL of the purified phage solution. The lysate was incubated overnight at 37 °C and was then pelleted at 12,500× *g* for 15 min. The resulting supernatant was filter sterilized through a 0.2 μm filter. A double agar overlay method was used for titration to enumerate the phage in each supernatant. *E. coli* (ATCC 43895) cells were cultured to log phase, then diluted to an OD_620_ of 0.8 to 1.0. Serial dilutions of each phage solution were performed, and 0.2 mL of the *E. coli* (ATCC 43895) cells were mixed with 10 μL of the phage solution. The cells were incubated with the phage for ten minutes before adding 3 mL LBM soft overlay or top agar and pouring the mixture onto an LBM underlay (bottom agar). Phage plaques were then enumerated to obtain the plaque-forming units per mL (PFU/mL). Bacteriophage isolates were amplified to titers >10^8^ PFU/mL [18]. Dimethyl sulfoxide (DMSO) was added to each bacteriophage stock solution until a final concentration of 7% volume to volume was reached. Bacteriophage stocks were then stored at −80 °C [19].

### 2.2. Bacteriophage Morphology Determination

Bacteriophages were concentrated and purified with Polyethylene Glycol (PEG) [20]. Briefly, DNase I and RNase were added to a flask containing 5 mL of each phage supernate to a final concentration of 1 µg/mL and incubated at 26 °C for 30 min. NaCl was added to a final concentration of 1 M and placed on ice or 1 h. In order to remove bacterial debris, samples were centrifuged at 11,000× *g* for 10 min at 4 °C. Maintaining a temperature of 4 °C, PEG 8000 was added to a final concentration of 10%. Tubes were rocked at 4 °C for 1.5 h. Tubes were centrifugated at 11,000× *g* for 10 min at 4 °C to precipitate the phage. The supernatant was carefully removed from the pellet and 1 mL of SM buffer was added to the pellet. Phage pellets were resuspended in 1 mL SM buffer and were stained with 2% aqueous (*w*/*v*) uranyl acetate adjusted to pH 4.2 and examined with a Philips EM 301 Transmission Electron Microscope operated at 60 kV. Bacteriophages were observed at high magnification (×71,000) [20]. The images were edited with ImageJ software version 1.46r.

### 2.3. Bacterial Culture for Microplate Study

Pathogenic *Escherichia coli* O157:H7 (ATCC 35150) was obtained from ATCC. Stock cultures were prepared by resuspending cells onto skim-milk media (Difco, Becton-Dickenson Labs, Annapolis, MD, USA) and stored at −25 °C. *E. coli* (ATCC 35150) were grown in tryptic soy broth (TSB, Difco, Becton-Dickenson Labs, MD, USA), supplemented with 5 mM of Magnesium sulfate (MgSO_4_, Fisher Scientific, Branchburg, NJ, USA) and Calcium chloride (CaCl_2_, Fisher Scientific, NJ, USA). All (working stock) cultures were held at refrigeration temperature (4 °C) for short term storage and −25 °C for long term storage.

### 2.4. Bacteriophage Spot Assay and Bacteriophage Cocktail Titer

Bacteriophages were spot-tested against both O157:H7 and non-O157:H7 Shiga toxin-producing *E. coli* (STEC) to validate the specificity of the isolated phages (Table 1). Bacteriophage titer was measured before the study for each bacteriophage used in the experiments to measure phage activity. The host strain for all the bacteriophages for the phage titer was *E. coli* (ATCC 35150). Phage titer ranged approximately 10^9^ PFU/mL for the phage cocktail.

### 2.5. Microplate Turbidometric Growth Inhibition Assays and Plate Count Study

*E. coli* (ATCC 35150) was used as the indicator microorganism for the microplate inhibition assay. An equal volume of C14 s, L1, LL15, and V9 phages were mixed in a sterile tube to obtain a phage cocktail. Fresh sterile TSB and TSB combined with 100 µL of *E. coli* (ATCC 35150) were used as a positive control treatment. TSB with a phage cocktail acted as a negative control to prove that bacteriophages do not contribute to turbidity at 660 nm. A volume of 100 µL of overnight-grown *E. coli* (ATCC 35150) (~1 × 10^9^ CFU/mL) was inoculated in TSB broth, and distributed to wells in a 96-well flat-bottom microtiter plate (Thermo Fisher Scientific). A bacteriophage cocktail (100 µL) was added and mixed by aspiration using a multi-channel micro-pipette contributing to an MOI (multiplicity of infection) of 1. The settings for the turbidity analysis using a microplate reader (BioTek, Synergy 4) were developed from Vijayakumar, PP, and P.M. Muriana (2015) [16]. The settings for the turbidity analysis were as follows: temperature: 37 °C (range: 36.5–37 °C); number of flashes: 1; measurement mode: absorbance; measurement wavelength: 660 nm; start kinetic (run: 3:00:00, interval 00:30:00); shake duration (orbital): 10 s (s); shake intensity: medium; total measurement time: 24 h (h); and unit: optical density (OD). The 96-well plate was sealed with a microplate lid to prevent evaporation of the liquid and well-to-well contamination. The OD_660_ values obtained were plotted against time and were used to illustrate the antimicrobial activity of the phage cocktail preparations against *E. coli* (ATCC 35150). Samples from the microplate wells were collected every three hours in a sterile manner for both control and treatment for up to 12 h from different wells. The obtained samples were then diluted (1:10) using peptone water and plated on pre-made tryptic soy agar (TSA, Difco, Becton-Dickenson Labs, Maryland, USA) plates supplemented with 5 mM Calcium chloride and Magnesium sulfate in triplicate. The plates were then incubated overnight at 37 °C, and the colonies were counted.

### 2.6. Microplate Turbidometric Growth Inhibition Assays of Bleach/Peroxyacetic Acid (Sanidate 5.0) Treated Bacteriophage Cocktail

The bacteriophage cocktail was exposed to 100-ppm bleach (Clorox regular, Oakland, CA, USA) water for 0, 1, 2, and 3 h. Fresh bleach water (100-ppm free chlorine) solution was prepared using sterile double-distilled water. The available chlorine in the bleach water was verified using chlorine test strips (Franklin machine products, Lumberton, NJ, USA). A volume of 500 µL bacteriophage cocktail (10^9^ PFU/mL) was added to 5 mL of 100-ppm sterile bleach water, and the mixture allowed to sit at room temperature for 3, 2, 1, and 0 h. Sterile deionized water (10 µL) was supplemented with Sodium thiosulfate (Na_2_S_2_O_3_, Fisher Scientific, NJ, USA) (0.5 mg/mL) before adding the 100 µL of bleach-treated phages to the broth in order to eliminate the effect of bleach on the pathogen from the results. A volume of 100 µL *E. coli* O157:H7 (10^9^ CFU/mL) was added to appropriate wells contributing to an MOI of 1. The microplate study was conducted as previously described, and the OD_660_ values were plotted against time and were used to illustrate the antimicrobial activity of bleach-treated phage cocktail preparations against *E. coli* (ATCC 35150). The experiment was repeated with organic sanitizer SaniDate 5.0 (100-ppm peroxyacetic acid, Biosafe systems, CT, USA) to determine the ability of the cocktail to survive the organic sanitizer. A study with *E. coli* (ATCC 35150) alone in each sanitizer (containing 100-ppm of the active ingredient) was performed to determine the pathogen’s ability to survive the sanitizers.

### 2.7. Spot Assay of Bacteriophages following a Mild Heat Stress

The effect of mild heat exposure on the bacteriophage’s ability to infect *E. coli* O157:H7 (ATCC 35150) was evaluated. Temperatures were chosen to mimic the environmental conditions often experienced in fresh produce processing. Phage preparations (150 µL) were transferred into a sterile Eppendorf tube and placed in a heating block (Techne, DRI-Block, DB-2 A) at 35, 45, and 55 °C; range ±0.2 °C in triplicates. An Eppendorf tube containing TSB and a temperature probe acted as a control and was also used for monitoring the temperature. The first phage tube preparations were heated to 35 °C, immediately removed from the heating block, and were placed in an ice bath. The second phage tube preparation was allowed to sit at 35 °C for 15 min and was then placed in the ice bath. A similar procedure was repeated at temperatures of 45 and 55 °C. All the samples were then spotted along with a control (no temperature treatment) onto a lawn of *E. coli* (ATCC 35150).

### 2.8. Statistical Analysis

Generalized estimating equations with Huber-White standard error estimates were used to approximate the mean response for all outcomes. Studies were considered as independent clusters with repeated measures on wells. Because of the non-linear trends of the response over time, time was treated as a categorical factor, and Tukey’s HSD (Honest Significant Difference) was used to compare treatments at each time point.

## 3. Results

### 3.1. Bacteriophage Screening, Isolation, and Amplification

**Figure 1:**
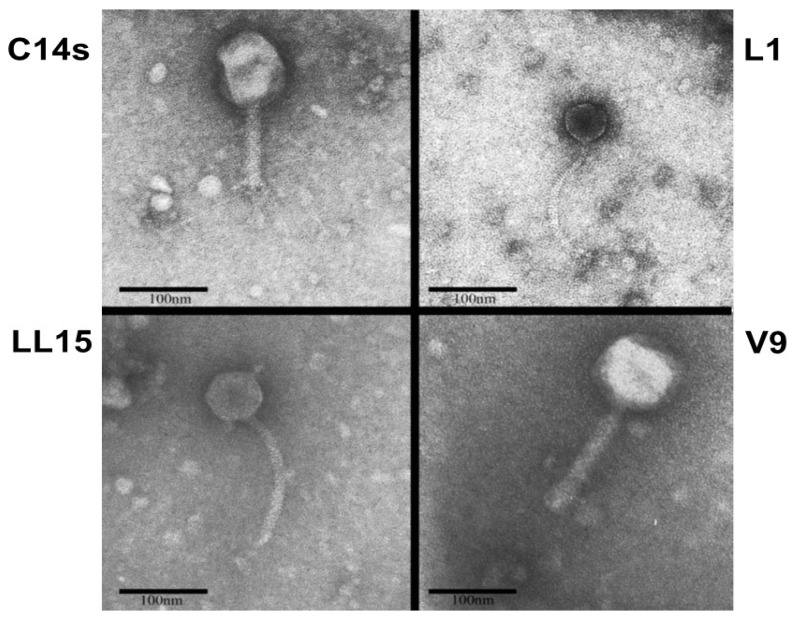
Electron microscopic images of the isolated bacteriophages from bovine origin.

Four wild bacteriophages (C14 s, L1, LL15, and V9) with lytic activity for *E. coli* O157:H7 (ATCC 43895) were isolated from dairy calf feces (Auburn College of Veterinary Medicine dairy herd). Examination by transmission electron microscopy (TEM) revealed phenotypic morphology for the four bacteriophages (Figure 1). Bacteriophages L1 and LL15 appear as typical members of the family Siphoviridiae of dsDNA bacteriophages [22], similar to the T5 and T1 morphotype [23,24,25]. Bacteriophages C14 s and V9 appear as members of the family Myoviridiae of dsDNA bacteriophages [22], similar to the T4 morphotype and 01 morphotype, respectively [23,24,26].

### 3.2. Microplate Growth Inhibition Assay and Plate Count Study of Bacteriophage Cocktail against E. coli O157:H7

**Figure 2:**
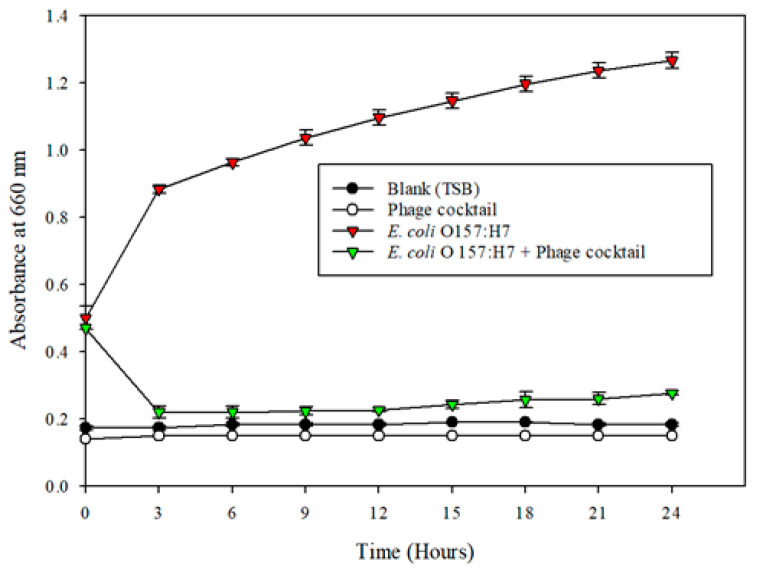
Microplate growth inhibition assay showing the activity of bacteriophage cocktail against *E. coli* O157:H7 (ATCC 35150). The data points represent the means of triplicate replication, and the error bars represent the standard deviations of three independent experiments. The bacteriophage cocktail reduced the population of *E. coli* O157:H7 (ATCC 35150) significantly (*p* < 0.05) compared to the control.

Positive controls of *E. coli* O157:H7 demonstrated a typical growth pattern. Significant inhibition of the pathogen was observed in the treatment wells containing the bacteriophage cocktail (Figure 2); thus, the bacteriophage cocktail preparation decreased the growth of *E. coli* (*p* < 0.01) in a controlled environment. The percentage reduction of *E. coli* in the presence of the bacteriophage cocktail at the end of three hours was 99.99%. The bacteriophage cocktail maintained the 5-log reduction (99.99%) until the end of 6 h, after which there was a subsequent decrease in the reduction percentage to 4-logs (9 h) and 2-logs (12 h), achieving 99.93% and 95.81% reduction respectively (*p* < 0.01) (Table 2).

### 3.3. Microplate Growth Inhibition of Bleach/SaniDate 5.0 Treated Bacteriophage Cocktail against E. coli O157:H7

**Figure 3:**
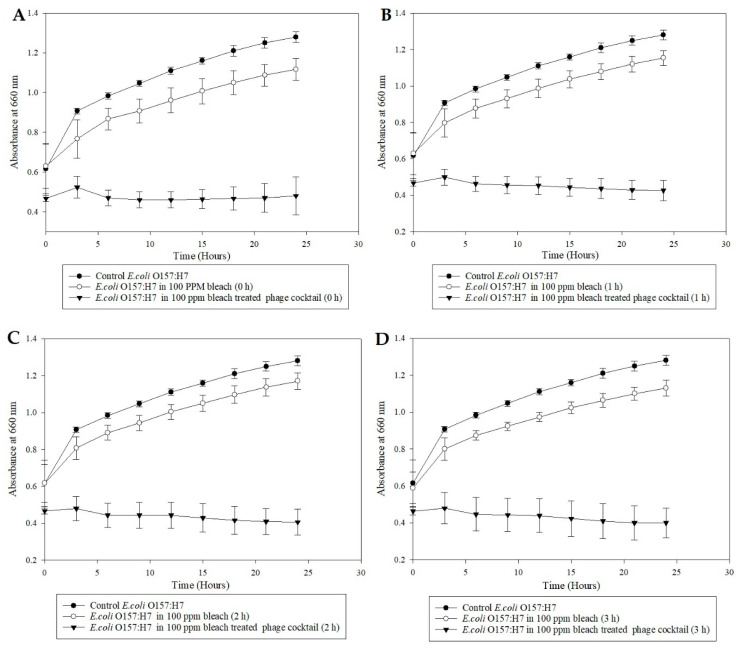
Microplate growth inhibition assay showing the activity of *E. coli* O157:H7 (ATCC 35150) in the presence of 100 parts per million (ppm) bleach and 100-ppm bleach treated phages at (**A**) 0 h, (**B**) 1 h, (**C**) 2 h, and (**D**) 3 h. The data points represent the means of triplicate replication, and the error bars represent the standard deviations of three independent experiments. The 100-ppm bleach treated bacteriophage cocktail significantly (*p* < 0.05) reduced the population of *E. coli* O157:H7 (ATCC 35150) at 0, 1, 2, and 3 h compared to the controls.

**Figure 4:**
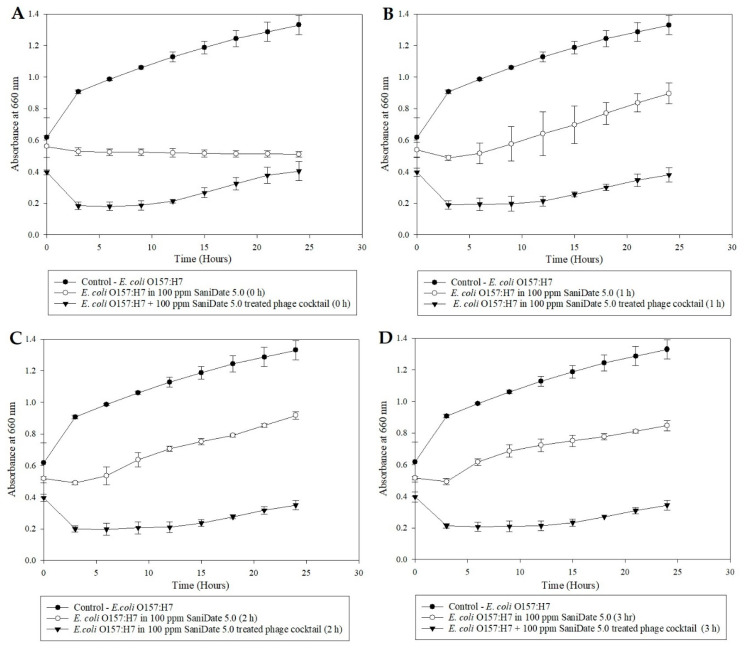
Microplate growth inhibition assay showing the activity of *E. coli* O157:H7 (ATCC 35150) in the presence of 100-ppm SaniDate 5.0 and 100-ppm SaniDate 5.0 treated phages at (**A**) 0 h, (**B**) 1 h, (**C**) 2 h, and (**D**) 3 h. The data points represent the means of triplicate replication, and the error bars represent the standard deviations of three independent experiments. The 100-ppm SaniDate 5.0-treated bacteriophage cocktail significantly (*p* < 0.05) reduced the population of *E. coli* O157:H7 (ATCC 35150) at 0, 1, 2, and 3 h compared to the controls.

A microplate inhibition assay was performed to study the efficacy of a bleach-treated bacteriophage cocktail against *E. coli* over time. Despite the exposure to bleach, the phage cocktail showed inhibition against the indicator microorganism (Figure 3) with a significant reduction in the OD (*p* < 0.05). At the same time, the pathogen without the phage cocktail demonstrated a classic growth curve, indicating that 100-ppm bleach had little to no effect against the pathogen (Figure 3). In 2002, Vijayakumar and Wolf-Hall studied the bactericidal concentration of bleach on different strains of *E. coli.* They determined that the minimum bactericidal concentration of bleach to be effective against the pathogen was between the range of 1.7–2.5% available chlorine in the water. It was also concluded that certain strains of *E. coli* were more resistant to bleach than others [27], which is comparable to the growth of the pathogen in the presence of 100-ppm bleach (Figure 3) in the current study. In the case of the organic sanitizer, 100-ppm SaniDate 5.0 at 0 h resulted in a statistically significant pathogen inhibition. However, as exposure time increased, the pathogen recovered in the sanitizer (Figure 4). Alternatively, the SaniDate 5.0-treated phage cocktail gave a consistent reduction in the population of *E. coli* compared to control, irrespective of being treated at different time intervals in the presence of the sanitizer (Figure 4). These results indicated the phage cocktail’s ability to survive and contribute to the reduction of *E. coli* O157:H7, despite being exposed to the commercially used sanitizers. These experiments demonstrate the potential of using the bacteriophage cocktail in combination with sanitizers, especially when washing produce where the combination can act as a hurdle technology to reduce the contamination of *E. coli* O157:H7 on fresh produce.

### 3.4. Spot Assay of Bacteriophages following a Mild Heat Stress

**Figure 5:**
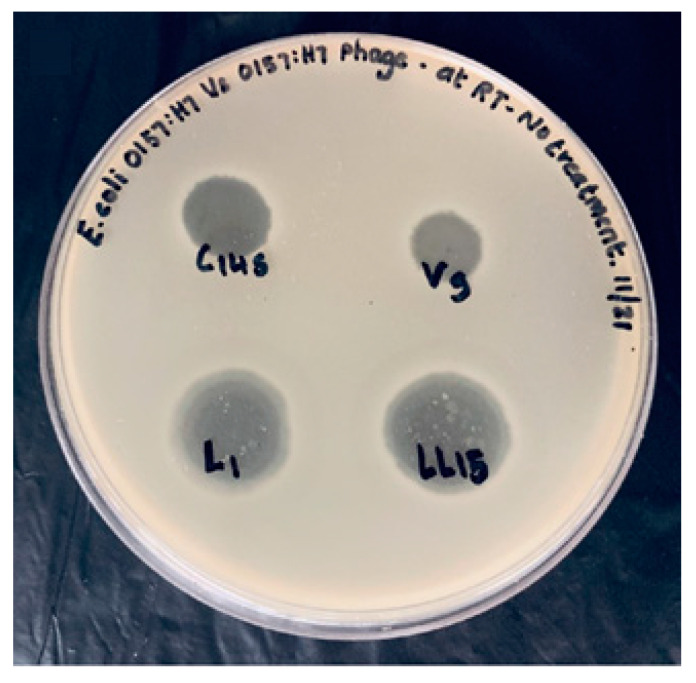
Double agar plate showing bacteriophage spot assay (C14 s, V9, L1, and LL15) against *E. coli* O157:H7 (ATCC 35150).

**Figure 6:**
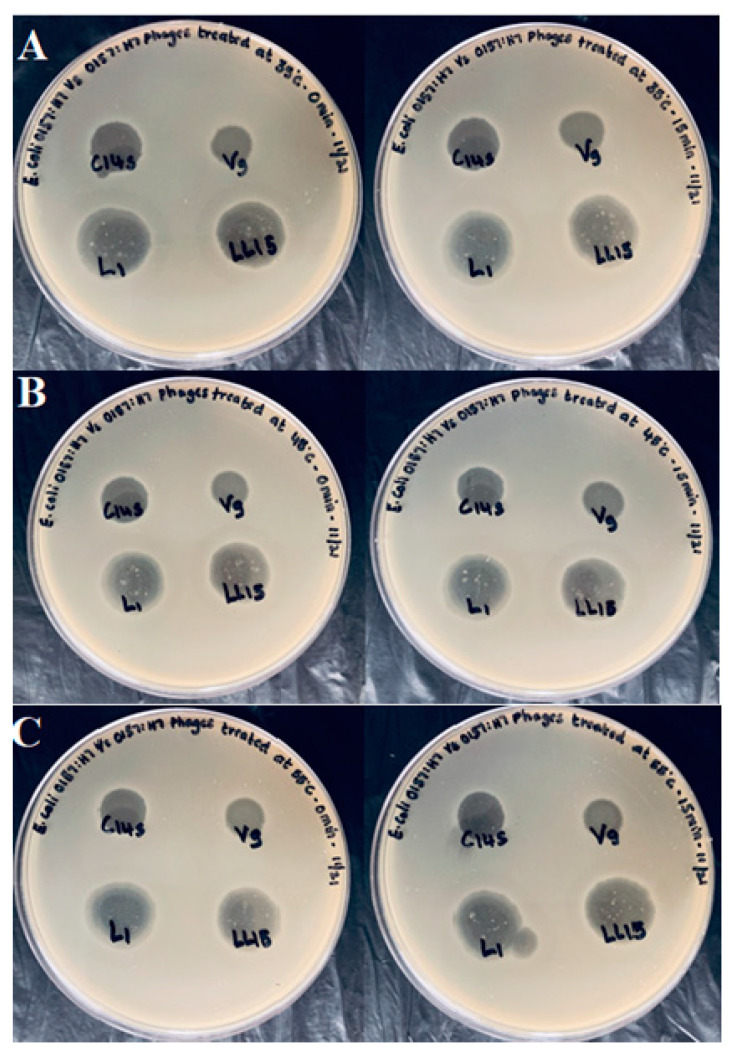
Double agar plate showing spot assay of heat-stressed bacteriophages against *E. coli* O157:H7 (ATCC 35150) at (**A**) 35, (**B**) 45, and (**C**) 55 °C at 0 and 15 min respectively.

Bacteriophage preparations were examined for heat tolerance, as an indication that the preparations would survive warm environment applications, especially those used on produce during wash treatments. No difference in bacteriophage activity was observed when centrifuged/heat treated bacteriophage preparations were compared to filter-sterilized preparations with no heat treatment (Figure 5). In subsequent heating trials, temperatures were increased to 45 and 55 °C for 0–15 min, with similar results (Figure 6). Temperature not only plays a vital role in survivability, but also helps in attachment, penetration, and multiplication of bacteriophages [28]. The ability to survive mild heat stress demonstrates that these bacteriophages may be added to a produce wash and still retain their ability to infect and reduce the population of *E. coli*.

## 4. Discussion

Bacteriophages, specifically those infecting *E. coli* O157:H7, were successfully isolated and identified from bovine feces. Bacteriophages were utilized in combination (cocktail) to eliminate the potential for developing a phage-resistant *E. coli* O157:H7 mutant against individual phages. The initial microplate study verified the efficacy of the bacteriophage cocktail against the pathogen, which indicates its potential to be used as an antimicrobial. The following study demonstrated that the bacteriophage cocktail could survive 100-ppm free chlorine and 100-ppm peroxyacetic acid. Allwood et al. (2005) studied the ability of F-specific RNA coliphage to survive 50-ppm concentration of free chlorine maintained at different temperatures (4, 25, and 37 °C) for up to 28 days. The study demonstrated that F-RNA coliphage had a higher survival rate for 7 to 14 days in 50-ppm chlorine-treated water at all temperatures. Since the coliphages were relatively resistant to chlorine, they can then be indicators for virological risk associated with products exposed to high concentrations of chlorine-based sanitizers [29]. The ability of bacteriophages to survive in the presence of these sanitizers opens new avenues for bacteriophage and sanitizers to be utilized, in combination, by the produce industry.

The post-harvest wash process is considered a critical control point in the fresh produce processing industry for removing field-accrued contamination [30]. It is well known that the produce industries rely on wash water sanitation to reduce the microbial load, maintain quality, and give an extended shelf life to products [31]. Many alternative techniques have encouraged the food industries to move away from bleach, due to various issues with maintaining its efficacy, and health problems that are associated with employing this longstanding disinfectant [31]. The current study also demonstrated the efficacy issue related to long term sanitizers. The sanitizer solution containing Sanidate 5.0 had a lower disinfectant effect than the one at 0 h when left to sit at room temperature for 1–3 h. With bleach, the 100-ppm concentration had little to no effect on the pathogen’s population.

Thus, continuous monitoring of sanitizer concentration is the most critical component of the produce wash procedure [32]. In contrast, the bacteriophage cocktail gave a consistent reduction in *E. coli* O157:H7 populations from 0–3 h irrespective of being exposed to these sanitizers compared to control. Therefore, if a deviation occurs, concerning the concentration of the sanitizer present during the produce wash with a bacteriophage cocktail/sanitizer combination, the phages would still be able to reduce the pathogen population resulting in a safe product.

Dunk/dip/immersion tank washing for produce is one of the most high-risk practices requiring investigation in the produce industry. Several foodborne outbreaks related to fresh produce have been traced back to improper post-harvest handling. Thus, poor wash water quality and improper sanitation may serve as a vector for contaminating produce when washed in dunk tanks. For this reason, bacteriophages are a promising antimicrobial for use in the food system as an effective bio-preservative, especially in ready-to-eat produce such as spinach, lettuce, and other leafy greens. Due to their ability to act as a natural antimicrobial, they can be integrated as a part of a multi-level sanitation process with commercially used sanitizers to eliminate pathogens of concern. Crude screening methods, such as plaque and microplate assays, would not be sufficient to forecast their effectiveness in a more complex system such as a produce wash. Therefore, future studies involving a wash system with a bacteriophage and sanitizer cocktail are necessary to understand their true potential in real-world environments.

## Figures and Tables

**Table 1:** Bacteriophage spot assay of O157:H7 and non-O157:H7 STEC’s.

*E. coli*-ATCC Number	Source	Serotype	Gene	Spot Assay Score *				
				C14 s	V9	L1	LL15	Phage Cocktail
35,150	Human feces	O157:H7	stx1, stx2, and eaeA	4	3	4	4	4
43,895	Raw hamburger meat	O157:H7	stx1, and stx2	4	3	4	4	4
2196	Stool sample	O26:H11	stx1, stx2, and eaeA	2	0	0	0	2
2215	-	O103:H11	stx1, and eae	0	0	0	1	1
2193	Stool sample	O45:H2	stx1, and eae	0	0	0	0	0
2219	Stool sample	O121:H19	stx2, and eae	0	0	0	0	0
2440	Human	O111	stx1, stx2, and eae	0	0	0	0	0

*—The scores represent the visual assessment of plaques on the spot assay. The system was adapted from Turner et al. [21] (2012).

**Table 2:** Reduction of *E. coli* O157:H7 (ATCC 35150) population in the presence of bacteriophage cocktail (C14 s, V9, L1, and LL15). A significant reduction (*p* < 0.05) in the population of *E. coli* O157:H7 (ATCC 35150) was observed between control and treatment.

Hours	Bacterial Populations (Log CFU/mL)		Log Reduction
	Control	Treatment	
3	8.99	3.81	5.18
6	8.08	4.68	3.40
9	9.14	5.68	3.46
12	9.31	7.64	1.67
